# Disruption of the MyoD/p21 Pathway in Rhabdomyosarcoma

**DOI:** 10.1080/13577149778218

**Published:** 1997-12

**Authors:** Michael Weintraub, Thea Kalebic, Lee J. Helman, Kishor G. Bhatia

**Affiliations:** Pediatric Branch National Cancer Institute National Institutes of Health Building 10, Room 13N240 Bethesda MD 20892 USA

## Abstract

*Purpose*. Rhabdomyosarcoma (RMS) is an embryonal tumor thought to arise from skeletal muscle cells that fail to differentiate terminally. The majority of RMSs express MyoD, a protein essential to the differentiation of skeletal muscle. It was recently shown that during myogenesis, MyoD activates the expression of the cyclin-dependent kinase inhibitor (CDKi), p21, which itself plays a critical role in normal muscle development. To investigate the integrity of the MyoD/p21 pathway in RMS, we analyzed p21 and its relationship to MyoD expression in RMS.

*Methods*. A panel of RMS samples was assembled from primary biopsies and from cell lines. Integrity of p21 was analyzed by single-strand conformation polymorphism (SSCP) and sequencing. Expression of p21 and MyoD was determined by Northern blot analysis, and the ability of exogenous p21 to arrest the cell cycle of RMS cell line was determined by transfection studies.

*Results*. Our analysis indicates that although p21 is wild type in RMS, there is an inverse correlation between the levels of p21 and MyoD in these tumors. Tumors that express significant amounts of MyoD fail to express p21. This does not appear to be the result of mutations within the potential CACGTG sites present in the p21 promoter region or in the coding region of p21. An additional group of RMSs express very high levels of p21 but express little, if any, MyoD. Furthermore, RD, a RMS cell line which expresses high levels of endogenous p21, undergoes withdrawal from the cell cycle following forced expression of p21, suggesting that the pathway which would lead to G_1_ arrest from endogenous p21 activity is defective.

*Discussion*. These data suggest that the interaction between p21 and MyoD is defective in RMS although the precise nature of the defect remains to be elucidated.


*Sarcoma* (1997) **1**, 135–141

CARFAX

**ORIGINAL ARTICLE**

### Disruption of the MyoD/p21 pathway in rhabdomyosarcoma

MICHAEL WEINTRAUB, THEA KALEBIC, LEE J. HELMAN & KISHOR G. BHATIA

*Pediatric Branch, National Cancer Institute, Bethesda, USA*

**Abstract**

*Purpose.* Rhabdomyosarcoma (RMS) is an embryonal tumor thought to arise from skeletal muscle cells that fail to differentiate terminally. The majority of RMSs express MyoD, a protein essential to the differentiation of skeletal muscle. It was recently shown that during myogenesis, MyoD activates the expression of the cyclin-dependent kinase inhibitor (CDKi), p21, which itself plays a critical role in normal muscle development. To investigate the integrity of the MyoD/p21 pathway in RMS, we analyzed p21 and its relationship to MyoD expression in RMS.

*Methods.* A panel of RMS samples was assembled from primary biopsies and from cell lines. Integrity of p21 was analyzed by single-strand conformation polymorphism (SSCP) and sequencing. Expression of p21 and MyoD was determined by Northern blot analysis, and the ability of exogenous p21 to arrest the cell cycle of RMS cell line was determined by transfection studies.

*Results.* Our analysis indicates that although p21 is wild type in RMS, there is an inverse correlation between the levels of p21 and MyoD in these tumors. Tumors that express significant amounts of MyoD fail to express p21. This does not appear to be the result of mutations within the potential CACGTG sites present in the p21 promoter region or in the coding region of p21. An additional group of RMSs express very high levels of p21 but express little, if any, MyoD. Furthermore, RD, a RMS cell line which expresses high levels of endogenous p21, undergoes withdrawal from the cell cycle following forced expression of p21, suggesting that the pathway which would lead to $G_1$ arrest from endogenous p21 activity is defective.

*Discussion.* These data suggest that the interaction between p21 and MyoD is defective in RMS although the precise nature of the defect remains to be elucidated.

**Key words:** *rhabdomyosarcoma, cell cycle, MyoD, p53, differentiation.*

### Introduction

Rhabdomyosarcoma (RMS) is the most common soft tissue sarcoma in children and comprises 5–8% of all pediatric tumors.[1] This embryonal tumor is thought to arise from poorly differentiated mesenchymal cells, with morphologic and biochemical similarities to primitive skeletal muscle cells. These tumors behave in an aggressive fashion, have a high proliferative capacity and fail to differentiate into mature muscle cells.

The mechanisms involved in the regulation of skeletal muscle differentiation have been investigated intensively. The pathways which lead to terminal differentiation appear to be coupled to those which cause withdrawal from the cell cycle and, presumably, this allows cells to maintain a differentiated state. The closely related processes of terminal differentiation and growth arrest are tightly regulated through complex mechanisms involving cyclins, cyclin-dependent kinases (CDK's) and CDK inhibitors (CDKI's).[2] Basic helix–loop–helix (HLH) transcription factors in the MyoD family induce expression of skeletal muscle specific proteins and withdrawal from the cell cycle.[3] Recently, this process has been shown to be associated with the induction of p21 (WAF1/Cip1), [4,5] a potent CDKI. Furthermore, MyoD has been shown to induce the expression of p21 directly,[5] suggesting a direct relationship between terminal muscle differentiation and growth arrest.

An early event in the transformation process in RMS may be related to the dysregulation of the normally tightly coupled pathways of muscle differentiation and growth arrest. RMSs are known to express MyoD or other related HLH proteins,[6] yet retain their ability to proliferate and fail to differentiate terminally. It has been shown that MyoD derived from RMS cell lines is capable of binding to its cognate DNA sequences, but is unable to transactivate muscle-specific genes, suggesting that RMS cells are deficient in a factor required for MyoD activity.[7] Since MyoD activates the expression of p21 in normal developing muscle, and since we recognized the presence of potential HLH binding

Correspondence to: K. G. Bhatia, Pediatric Branch, National Cancer Institute, National Institutes of Health, Building 10, Room 13N240, Bethesda, MD 20892, USA. Tel: + 1 301 496 2321; Fax: + 1 301 480 5648; E-mail: bhatiak@pbmac.nci.nih. gov.

1357-714X/97/030135-07 $9.00 © 1997 Carfax Publishing Ltd

**Table 1.** *Rhabdomyosarcoma cell lines and tumors*

| | Sample | Histology | Expression of MyoD1 | Expression of p21 | p53 status |
|---|---|---|---|---|---|
| Cell lines | RD | Embryonal | + | + + + | Mutation Arg Trp Codon 248 |
| | RH18 | Mixed Alveolar/Embryonal | + | + + + | WT |
| | RH28 | Alveolar | ND | ND | WT |
| | RH30 | Alveolar | + + + | + | Mutation Arg Ser Codon 280 |
| | CTR | Embryonal | + + + | + | Deletion 4 bP Codons 219–220. |
| Tumors | PO49 | Alveolar | + | + | WT |
| | PO50 | Alveolar | + + + | + + + | WT |
| | PO51 | Alveolar | ND | ND | WT |
| | PO52 | Alveolar | + | + | WT |
| | 9003 | Embryonal | ND | ND | WT |
| | I-12 | Alveolar | + + + | + | ND |
| | 5700 | ND | + | + + + | WT |
| | SWMC | Embryonal | ND | ND | Both alleles deleted |
| | I-174 | Alveolar | + | + | ND |

Abbreviations: ND, not determined; WT, wild type; + + +, high expression; +, low expression.

sites in the promoter sequence of p21,[8] it was of interest to determine whether expression of MyoD coincides with expression of p21 in RMS. In addition, we also investigated the integrity of the p21 $G_1$ arrest pathway in RMS by analyzing the primary structure of p21 in RMS and the ability of exogenous p21 to cause forced withdrawal of RMS cells from the cell cycle.

### Patients and methods

### *Cell lines and tumor samples*

Tumor histology and p53 status are described in Table 1. All tumor tissues were obtained from the Cooperative Human Tissue Network and had been confirmed to contain viable tumor. Tumor samples PO49, PO50, PO51, PO52, 5700, I-174 and I-12 are all alveolar RMSs. SWMC and 9003 are embryonal RMSs. RD is a human embryonal RMS cell line obtained from the American Type Culture Collection (ATCC).[9] RH18, RH28 and RH30 are human RMS cell lines established from mixed embryonal/alveolar (RH18) and alveolar (RH28, RH30) RMS and obtained from Dr Peter Houghton (St Jude Children's Research Hospital). CTR is a human embryonal RMS cell line established and supplied by C. P. Reynolds (Department of Pediatrics, University of Southern California). Cells were grown at 37°C in 95% air/5% $CO_2$ in RPMI 1640 containing 10% heat-inactivated fetal calf serum (FCS), 2 mM L-glutamine, 50 $\mu g\ ml^{-1}$ penicillin and 50 $\mu g\ ml^{-1}$ streptomycin.[10] All tissue culture products were obtained from Bio-Whittaker (Walkersville, MD, USA).

### *RNA isolation and Northern analysis*

Total cellular RNA was extracted from cell lines and tumor specimens as previously described.[11] RNA was quantitated using UV spectrophotometry at $A = 260$ nm. Equal amounts of RNA were electrophoresed in a 1.2% agarose/2.2% formaldehyde gel, transferred to a nylon membrane and hybridized with $^{32}$P-labeled (Random prime labeling: Prime-It, Stratagene, La Jolla, CA, USA) probes for p21 cDNA[12] and for Myo D.[13]

### *Plasmid constructs*

The pCEP4 expression vector (Invitrogen, San Diego, CA, USA) was used to prepare constructs containing the following cDNAs as previously described:[12] (1) full-length wild-type p21 cDNA; (2) full-length p21 cDNA in an anti-sense orientation; (3) a mutant full-length p21 cDNA, with a mutation at codon 63 (Phe to Leu).

### *Transfections*

p21 sense, p21 anti-sense or mutant p21 constructs were transfected into the RMS cell line RD using calcium phosphate precipitation as previously described.[14] Transfection with vector alone served as control. All transfection experiments were done in triplicate. Briefly, $5 \times 10^5$ cells were plated in six-well tissue culture plates, incubated overnight at 37°C and washed with fresh medium 3 h prior to transfection. Plasmid (1 $\mu g$) and 4 $\mu g$ of salmon-sperm carrier DNA were used for transfection in each well. After transfection, the cells were incubated for 12 h at 37°C, washed and fed with fresh medium (RPMI 1640 supplemented with 10% FCS). After 24 h, transfectants were selected in medium containing hygromycin (500 $\mu g\ ml^{-1}$), and growth of colonies was monitored daily for a period of 2 weeks. The plates were then stained with crystal violet and the colonies were counted by two independent observers. In separate experiments, following transfection and selection of colonies in hygromycin-containing medium, cells were trypsinized and counted directly by the trypan blue exclusion method.

### *PCR–SSCP–p21*

PCR–SSCP analysis[15] was used to screen for mutations in the coding region of p21 as previously reported.[12] In addition, we designed primers to analyze the promoter region for p21 by SSCP,[8] specifically including a region which contains the sequence CAGCTG, a consensus binding sequence for HLH proteins (E box).[16] Briefly, DNA was extracted from cell lines and tissue samples of RMS using standard methods. The coding region of the p21 gene was amplified using oligonucleotide primers to generate four fragments(2.1, 2.2, 2.3, 3) (Fig. 1). The primers used were: region 2.1: WAF 1, 5′-AGAGGAGGCGCCATGTCAGAA; WAF 1.1-3R, 5′AGGTAGAGCTTGGGCAGGCC; region 2.2: WAF 1.25F, 5′-CGAGACACCACTG-GAGGGTG; WAF 1.2R, 5′-CTTCAGCCT-GCTCCCCTG; region 2.3: WAF 2.3.5, 5′-TGGACCTGTCACTGTCTT; WAF 2.3.3, 5′-TGAGAATCCTGGTCCCTT; Region 3: WAF 3b5, 5′-GATTTCTACCACTCCAAA; WAF RT3, 5′-GGCCTTTGAG GCCCTCGCGCTT. Primers for the promoter region were: PROM-5, 5′-GAGGGAGGTCCCGGGCG; PROM-3, 5′-AATCCGCGCCCAGCTCCG. Radiolabeled PCR products were separated on a 1X MDE gel (Hydrolink, Malvern, PA, USA) at 12 W, constant power, at room temperature, for 12–18 h. A negative control and a blank PCR (reaction without template) were always included in the analysis to confirm the absence of exogenous contamination. PCR products with abnormal migration were sequenced using the Sequenase method (USB/Amersham, Cleveland, OH, USA).

### Results

### *Expression of p21 and MyoD*

We first sought to determine whether MyoD expression in RMS tumors parallels p21 expression as would be predicted. Ten samples of RMS (including four cell lines and six fresh tumor biopsies) were analyzed for expression of p21 and MyoD by Northern blot analysis. Expression of p21 was highly variable (Fig. 2). The cell lines RD and RH18, and tumors 5700 and PO50, expressed high levels of p21, whereas the cell lines CTR and RH30, and tumor samples I-12, I-174, PO49 and PO52, expressed low levels of p21. Expression of MyoD was also variable. High levels of expression were seen in cell lines RH30 and CTR, and in tumor samples I-12 and PO50. In contrast, low levels of MyoD expression were seen in cell lines RD and RH18, and in tumor samples I-174, PO49, PO52 and 5700 (Fig. 2). Thus, in the majority of the samples, there was an inverse relationship between expression of p21 and MyoD, such that cell lines and tumors which expressed high levels of MyoD were found to express low levels of p21. For comparison, in normal, terminally differentiated adult skeletal muscle (Fig. 2, lane 1), modest levels of MyoD expression are associated with high levels of p21 mRNA expression. In contrast, tumors with similar, or higher, MyoD expression levels (samples

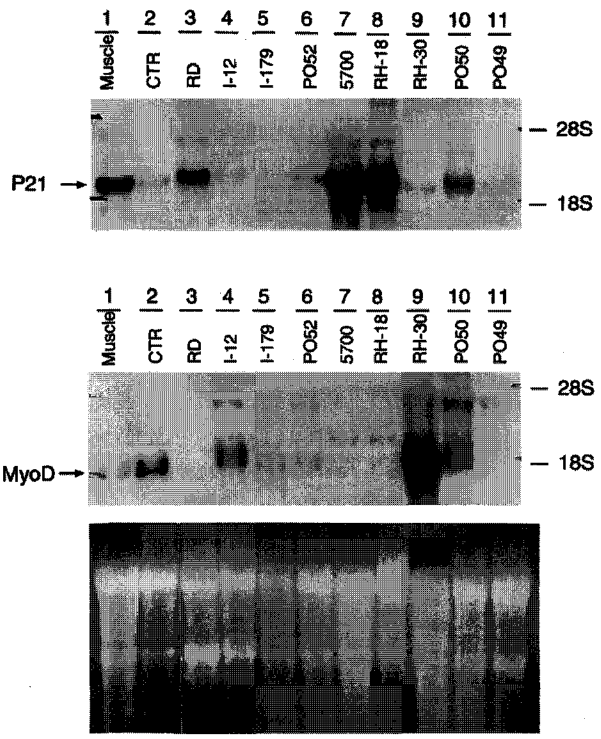

**Fig. 1.** *Structure of the coding region for p21 and regions used for amplification in SSCP analysis. Abbreviations: aa, amino acid; bp, base pair; WAF = p21.*

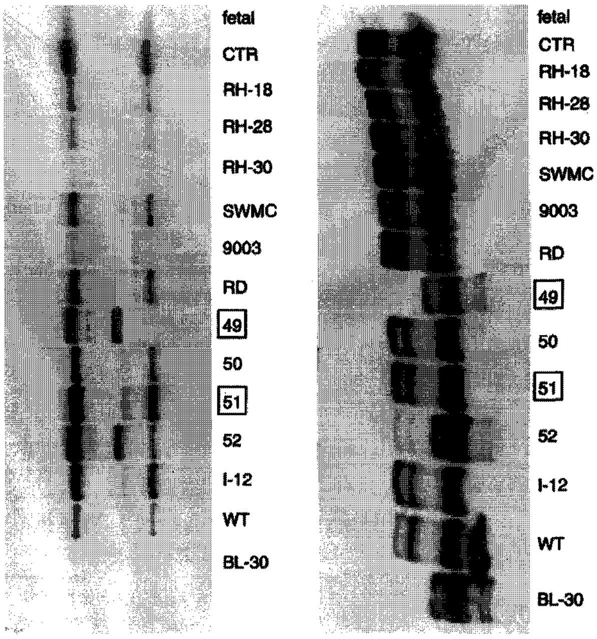

**Fig. 2.** *Northern blot analysis of the expression of p21 and MyoD in RMS tumors and cell lines: (a) expression of p21; (b) expression of MyoD; (c) ethidium bromide stained gel for comparison of RNA amounts.*

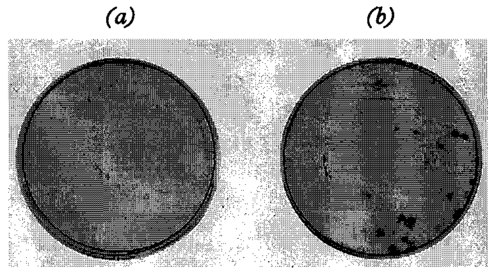

**Fig. 3.** *SSCP analysis for the coding region of p21: (a) region 3; (b) region 2.2. Samples PO49 and PO52 show an aberrant conformer in both regions. Abbreviation: WT, wild type.*

RH30, CTR, PO52, I-12, Fig. 2), express low levels of p21. A second group of RMS tumors and cell lines expresses high levels of p21 but no detectable levels of MyoD mRNA.

*SSCP analysis*

Since high levels of p21 mRNA expression were found in several cell lines and tumors, we sought to determine whether the primary structure of p21 was altered. SSCP analysis of the p21 coding region was performed in 12 RMS samples (four cell lines and eight tumors). Figure 3 demonstrates abnormal migration patterns in two samples from tumor DNA (PO49 and PO52, boxed). In both samples, there was an abnormal conformer in two regions: 2.2 and 3. Sequencing the two samples with region 2.2 abnormal conformers revealed a Ser to Arg substitution at codon 31. This substitution has been previously described as a polymorphism.[12,17] The same two samples were sequenced for region 3. Both were found to have a C→T transition at position 590 in the 3′ untranslated region of the p21 gene. This change has also been described previously[18] and is likely to be a polymorphism. No additional changes were detected. However, the presence of both the polymorphisms on the same allele has not been observed previously. Thus, no random mutations in the coding region of p21 were detected in this series of RMS cell lines and tumor samples. SSCP analysis of the promoter region for p21 (downstream of the TATA box, −30 bp from the transcription start site) also revealed no mutations (data not shown).

*Suppression of tumor cell growth/proliferation by p21*

Since we failed to detect alterations in the primary structure of the p21 gene in RMS, we sought to analyze the functional integrity of the p21 pathway in these tumors by forced expression of p21. Transfection of p21 into the RMS cell line, RD, caused a significant inhibition of cell colony formation as measured in a clonogenicity assay (Fig. 4 and Table 2). RD cells transfected with wild-type p21 showed

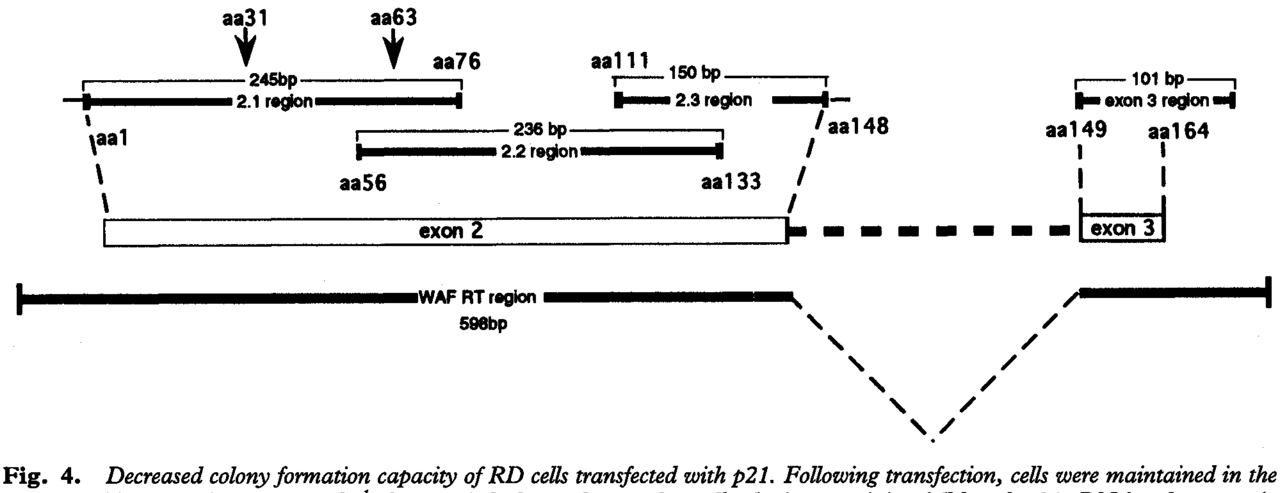

**Fig. 4.** *Decreased colony formation capacity of RD cells transfected with p21. Following transfection, cells were maintained in the presence of hygromycin (500 $\mu g\ ml^{-1}$, for a period of 3 weeks, to select cell colonies containing full length p21 cDNA or hygromycin plasmid. The experiment was performed in triplicate. Differences between replicates did not exceed 15%.*

**Table 2.** *Inhibition of cell proliferation in the RMS cell line RD by p21*

| Plasmid | Vector | p21 | p21 AS | Mutant p21 |
|---|---|---|---|---|
| (A) Cell number | 2.4 | 1.2 | 2.8 | 2.0 |
| (B) Colony number | 37 | 4 | 55 | ND |

Inhibition of cell proliferation in the RMS cell line RD by p21.

(A) Cell numbers ($\times 10^6\ ml^{-1}$) determined by direct cell counting.

(B) Number of colonies in a clonogenicity assay.

Abbreviations: vector, PCEP4 vector carrying hygromycin resistance gene only; p21, full-length wild-type p21 cDNA; AS, full-length wild-type p21 cDNA in anti-sense orientation; mutant, full-length p21 cDNA with a codon 63 mutation; ND, not done.

a 10-fold lower number of colonies ($n = 4$) when compared to the controls, represented by cells transfected with vector alone ($n = 37$) or with anti-sense p21 ($n = 55$). (Expression of the p21 protein in the transfected cells was confirmed by Western blot analysis (data not shown).)

Consistent with these results, the cells transfected with p21 showed a lower proliferative rate as determined by direct counting of cells derived from a pool of colonies after trypsinization (Table 2). Transfection with the mutant p21 had a lesser growth inhibitory effect than transfection with wild-type p21.

### Discussion

Several lines of evidence point to an important role played by the p21 protein in the process of normal muscle cell differentiation. It was initially shown that differentiated skeletal muscle cells express high levels of the hypophosphorylated form of the retinoblastoma (RB) protein,[19,20] and that loss of RB prevented $G_1$ arrest in differentiated muscle cells.[21,22] CDK-mediated inactivation of RB through phosphorylation causes cells to enter the cell cycle and proliferate.[23] By inhibiting the CDK's, p21 induces cell-cycle arrest and inhibits cell proliferation. Subsequent studies showed that, in developing skeletal muscle cells, arrest of cell proliferation is associated with increased expression of p21.[5] Furthermore, MyoD, a member of the family of myogenic basic HLH proteins involved in induction of muscle-differentiation specific genes, can induce expression of p21.[5] Consistent with this observation, we also identified several CACGTG sites in the regulatory region of p21.[8] Thus, p21 appears to be involved in the coupling of the two processes of cell proliferation arrest and terminal differentiation in skeletal muscle cells.[23]

Since RMS is a tumor of primitive skeletal muscle cells which undergo uncontrolled proliferation and fail to differentiate terminally into normal skeletal muscle, we chose to examine p21 status in these tumors. In normal skeletal muscle cells, expression of MyoD, and other muscle-specific genes, leads to terminal differentiation and growth arrest, associated with induction of p21 expression. Expression of MyoD is also seen in the majority of cases of RMS, irrespective of their histology.[24] Tonin *et al.*[25] investigated the expression of several muscle-specific genes in RMS of both embryonal and alveolar subtypes. Their data suggest that RMS tumors regardless of histological features expressed MyoD1. In the data we present (Table 1) the levels of MyoD1 expression vary in RMS-independent histology. Immunohistochemical analysis of MyoD1 expression in 33 RMS samples reported by Wang *et al.*[26] again demonstrated no significant differences in the expression of MyoD1 with respect to histological subtypes. Thus, it appears certain that the MyoD1 expression is an invariant marker of RMS. However, the expression of MyoD in RMS does not lead to proliferation arrest or to differentiation, implying that, in these cells, the MyoD pathway is functionally abnormal. This notion is supported by previous studies which showed that MyoD obtained from RMS was deficient in transactivation. Thus, the question arose whether MyoD from RMS can transactivate p21. We found that RMS cell lines and tumors that express very high levels of MyoD fail to express p21, suggesting an inability of MyoD in RMS to transactivate p21. RMS tumors that express p21, however, express low levels of MyoD. These results strongly suggest that the endogenous MyoD/p21 pathway in these cells is abnormal, and is unable to induce a cell-cycle arrest or promote differentiation. This hypothesis is supported by the finding that forced expression of wild-type p21 in the transfected RD cell line, which expresses a high endogenous level of p21, causes a marked inhibition of cell proliferation. This suggests that the endogenous p21 pathway in the RD cells is in some way compromised. Since the defect can be overcome by forced expression of exogenous p21, one may infer that pathways downstream of p21 are intact.

To determine if the defect in p21 in RD cells and in other RMS cells is the result of mutations in p21, we performed an SSCP analysis to detect mutation in the coding region of the p21 gene. Similar to studies in other tumors, the analysis revealed no mutations, suggesting that the putative defect in p21 in RMS is not due to primary structural alterations. It is possible that SSCP analysis may have missed some sequence changes;[27] however, our ability to detect polymorphic changes would suggest that this is not the case. Our data represent the first report on p21 mutation analysis in RMS, and the absence of p21 mutations in this tumor will need confirmation with larger sample numbers. However, the absence of mutations in the p21 gene is not unusual. Analyses of the p21 coding region in a large number of tumors of varying histologies have failed to reveal mutations in this gene,[18,28] with rare exceptions,[12] suggesting that a role for p21 in tumorigenesis may involve mechanisms distinct from mutations.[18]

Interestingly, we found an inverse correlation between the expression of p21 and the expression of MyoD. Cell lines and tumors expressing high p21 levels had a very low level of MyoD expression and, conversely, those cell lines and tumors with a high MyoD levels expressed little or no p21. This may imply that the normal process of cell-cycle arrest and terminal differentiation in skeletal muscle cells requires a cooperative action of p21 and MyoD. Indeed, studies in RMS cells have shown that these cells are deficient in a factor required for MyoD activity. Formation of heterokaryons between RMS cells and fibroblasts has been shown to restore the ability of the RMS cells to differentiate into muscle cells.[7] p21 may be the factor necessary for MyoD-

driven muscle cell differentiation, and its functional abnormality in RMS cells, as shown in our experiments, may explain the paradox of differentiation failure in the face of high MyoD expression in RMS. Our results suggest that the concurrent expression of both MyoD and p21 may be necessary for the integrity of the differentiation pathway in skeletal muscle cells. Thus, the mutually exclusive expression of MyoD and p21 in RMS suggests that there are at least two distinct pathways of RMS pathogenesis. In one group of tumors, p21 is expressed at high levels but, despite this, is unable to induce cell-cycle arrest, probably because the p21 in this group is compromised. Based on the results of our sequence analysis, it seems unlikely that this compromise is the result of a defect in the structural integrity of p21. However, several studies have suggested that there are at least two functionally distinct forms of cyclin–CDK kinase and p21 complexes. In one, the kinase is still active, while in the other, p21 inhibits the kinase activity.[29,30] Recent data (Harlow, personal communication, 1995) suggest that the formation of these two functional complexes may actually be based on the amount of p21 present in the complex. Thus, when present at high levels, p21 would form an inhibitory complex. In our experiments, over-expression of exogenous p21 in RMS cells that already express high levels of endogenous p21 did indeed cause growth arrest. Thus, one possible mechanism by which endogenous p21 causes growth arrest in RMS cells may be related to the stoichiometric composition of the cyclin–CDK–p21 complex, rendering the complex non-functional with respect to cell-cycle arrest. Increasing p21 levels in these cells by transfecting a p21 expression vector probably allows for a shift of the cyclin–CDK–p21 active complex to an inhibitory state—resulting in the arrest of proliferation. MyoD levels in this group of tumors are low to undetectable. This, in conjunction with a catalytically active endogenous CDK–cyclin–p21 complex, may also be related to the differentiation failure of these cells. A second group of RMSs express high levels of MyoD, but, in contrast to normal muscle, this expression fails to induce p21 and, presumably, to cause cell-cycle arrest. It is possible that the normal cascade of growth arrest and induction of terminal differentiation in skeletal muscle cells requires the cooperative action of both MyoD and p21, and it is this cooperative effect which is defective in RMS.

We found no association between the integrity of the p53 gene in these cells and the level of p21 expression. In both tumors and cell lines, p21 expression varied between high and low in samples with wild-type or mutant p53. This suggests that the expression of p21 in these cells is independent of p53. This observation is in agreement with studies in mice embryos where the expression of p21 during terminal differentiation is also p53 independent.[21]

p21 can inhibit cell proliferation by at least two distinct mechanisms: inhibition of CDK's and direct binding to PCNA.[31] Indeed, p21 has previously been shown to suppress growth of tumor cell lines.[32] Furthermore, forced expression of p21 has been shown to reverse the abnormal proliferation of embryonal fibroblasts transformed by various oncogenes.[33] Our results support the notion that p21 plays a critical role in the normal differentiation pathway of skeletal muscle cells. In addition, our study suggests that targeted over-expression of p21 in RMS cells may correct an important biochemical defect in these cells and thus make RMS a suitable tumor for new modalities of therapy with p21.

### Acknowledgement

This project was supported in part by the Cooperative Human Tissue Network, which is funded by the National Cancer Institute.

### References

1 Raney RB, Tefft M, Hays DM, *et al.* Rhabdomyosarcoma and the undifferentiated sarcomas. In: Pizzo PA, Poplack DG, eds. *Principles and practice of pediatric oncology*. 2nd edn. Philadelphia: Lippincott, 1993:769–94.

2 Pines J. Cyclins and cyclin-dependent kinases: theme and variations. *Adv Cancer Res* 1995; 66:181–212.

3 Olson EN. MyoD family: a paradigm for development? *Genes and Dev* 1990; 4:1454–61.

4 Parker SB, Eichele G, Zhang P, *et al.* p53-independent expression of p21$^{CiP1}$ in muscle and other terminally differentiating cells. *Science* 1995; 267:1024–7.

5 Halevy O, Novitch BG, Spicer DVB, *et al.* Correlation of terminal cell cycle arrest of skeletal muscle with induction of p21 by MyoD. *Science* 1995; 267:1018–21.

6 Scrable H, Witte D, Shimada H, *et al.* Molecular differential pathology of rhabdomyosarcoma. *Genes Chromosomes Cancer* 1989; 1:23–35.

7 Tapscott SJ, Thayer MJ, Weintraub H. Deficiency in rhabdomyosarcoma of a factor required for MyoD activity and myogenesis. *Science* 1993; 259:1450–3.

8 El-Deiry WS, Tokino T, Velculescu VE, *et al.* WAF1, a potential mediator of p53 tumor suppression. *Cell* 1993; 75:817–25.

9 McAllister RM, Melnyk J, Finkelstein J. Cultivation *in vitro* of cells derived from a human rhabdomyosarcoma. *Cancer* 1969; 24:520–6.

10 El-Badry O, Romanus J, Helman L, *et al.* Autonomous growth of a human neuroblastoma cell line is mediated by insulin-like growth factor II. *J Clin Invest* 1989; 84:829–39.

11 Chirgwin JM, Przybyla AE, Macdonald RJ, *et al.* Isolation of biologically active ribonucleic acid from sources enriched in ribonuclease. *Biochemistry* 1979; 18:5294–9.

12 Bhatia K, Fan S, Spangler G, *et al.* A mutant p21 cyclin-dependent kinase inhibitor isolated from a Burkitt's lymphoma. *Cancer Res* 1995; 55:1431–5.

13 Edmondson DG, Olson EN. A gene with homology to the myc similarity region of MyoD1 is expressed during myogenesis and is sufficient to activate the muscle differentiation program. *Genes and Dev* 1990; 3:628–40.
14 Gorman CM, Moffat LF, Howard BH. Recombinant genomes which express chloramphenicol acetyltransferase in mammalian cells. *Mol Cell Biol* 1982; 2:1044–51.
15 Orita M, Iwahara H, Kanazawa H, *et al.* Detection of polymorphisms of human DNA by gel electrophoresis as single strand conformation polymorphisms. *Proc Natl Acad Sci* 1989; 86:2766–70.
16 Tapscott SJ, Weintraub H. MyoD and the regulation of myogenesis by helix–loop–helix proteins. *J Clin Invest* 1991; 87:1133–8.
17 Chedid M, Michieli P, Lengel C, *et al.* A single nucleotide substitution at codon 31 (Ser/Arg) defines a polymorphism in a highly conserved region of the p53 inducible gene WAF1/CIP1. *Oncogene* 1994; 9:3021–4.
18 Shiohara M, El-Deiry WS, Makio W, *et al.* Absence of WAF1 mutations in a variety of human malignancies. *Blood* 1994; 84:3781–4.
19 Coppola JA, Lewis BA, Cole MD. Increased retinoblastoma gene expression is associated with late stages of differentiation in many different cell types. *Oncogene* 1990; 5:1731–3.
20 Endo T, Goto S. Retinoblastoma gene product Rb accumulates during myogenic differentiation and is deinduced by the expression of SV40 large T antigen. *J Biochem* 1992; 112:427–30.
21 Schneider JW, Gu W, Zhu L, *et al.* Reversal of terminal differentiation mediated by p107 in $RB^{-}/^{-}$ muscle cells. *Science* 1994; 264:1467–71.
22 Gu W, Schneider JW, Condorelli G, *et al.* Interaction of myogenic factors and the retinoblastoma protein mediates muscle cell commitment and differentiation. *Cell* 1993; 72:309–24.
23 Skapek SX, Rhee J, Spicer DB, *et al.* Inhibition of myogenic differentiation in proliferating myoblasts by cyclin D1-dependent kinase. *Science* 1995; 267:1022–4.
24 Dias P, Parham DM, Shapiro DN, *et al.* Myogenic regulatory protein (MyoD1) expression in childhood solid tumors: diagnostic utility in rhabdomyosarcoma. *Am J Pathol* 1990; 137:1283–91.
25 Tonin PN, Scrable H, Shimida H, *et al.* Muscle-specific expression in rhabdomyosarcomas and stages of human fetal skeletal muscle development. *Cancer Research* 1991; 51:5100–6.
26 Wang NP, Marx J, McNutt MA, *et al.* Expression of myogenic regulatory proteins (myogenin and MyoD1) in small blue round cell tumors of childhood. *Am J Pathol* 1995; 147:1799–810.
27 Sheffield VC, Beck JS, Kwitek AE, *et al.* The sensitivity of single-strand conformation polymorphism analysis for the detection of single base substitutions. *Genomics* 1993; 16:325–32.
28 Li YJ, Laurent-Puig P, Salmon RJ, *et al.* Polymorphisms and probable lack of mutations in the WAF1-CIP1 gene in colorectal cancer. *Oncogene* 1995; 10:599–601.
29 Fotedar R, Fitzgerald P, Rousselle T, *et al.* p21 contains independent binding sites for cyclin and cdk2: both sites are required to inhibit cdk2 kinase activity. *Oncogene* 1996; 12:2155–64.
30 Zhang H, Hannon GJ, Beach D. p21 containing cyclin kinases exist in both active and inactive states. *Genes and Dev* 1994; 8:1750–8.
31 Sherr CJ, Roberts JM. Inhibitors of mammalian $G_1$ cyclin-dependent kinases. *Genes and Dev* 1995; 9:1149–63.
32 Chen YQ, Cipriano SC, Arenkiel JM, *et al.* Tumor suppression by $p21^{WAFI}$. *Cancer Res* 1995; 55:4536–9.
33 Givol I, Givol D, Rulong S, *et al.* Over-expression of human $p21^{waf1/cip1}$ arrests the growth of chicken embryo fibroblasts transformed by individual oncogenes. *Oncogene* 1995; 11:2609–18.
